# Hydralazine Attenuates Lipopolysaccharide-Induced Murine Myocardial Dysfunction by Inhibition of Semicarbazide-Sensitive Amine Oxidase

**DOI:** 10.3390/antiox14121502

**Published:** 2025-12-14

**Authors:** Zejian Kuang, Hongjun Luo, Hui Li, Yongyin Zhou, Zhexuan Lin, Wenhong Luo

**Affiliations:** 1 Bio-Analytical Laboratory, Shantou University Medical College, Shantou 515041, China; s_zjkuang@stu.edu.cn (Z.K.); hjluosumc@stu.edu.cn (H.L.); hli@stu.edu.cn (H.L.); 18yyzhou@stu.edu.cn (Y.Z.); 2 Department of Critical Care Medicine, The Second Affiliated Hospital of Shantou University Medical College, Shantou 515041, China; 3 Department of Neurology, The First Affiliated Hospital of Shantou University Medical College, Shantou 515041, China

**Keywords:** sepsis-induced myocardial dysfunction, hydralazine, semicarbazide-sensitive amine oxidase (SSAO), oxidative stress, inflammatory infiltration

## Abstract

Sepsis-induced myocardial dysfunction (SIMD) is a fatal complication with limited therapeutic options. Semicarbazide-sensitive amine oxidase (SSAO) contributes to oxidative stress and leukocyte recruitment, yet its role in SIMD remains unexplored. This study investigates whether hydralazine, a potent SSAO inhibitor, protects against SIMD by evaluating the involvement of SSAO inhibition. Using a murine model of LPS-induced sepsis, hydralazine was administered 30 min post-injection. Over a 7-day observation period, survival rates, cardiac function (assessed by echocardiography), and myocardial injury (evaluated via plasma biomarkers including CK, CK-MB, LDH, and AST, alongside histopathology) were monitored. Additional analyses included measurements of oxidative stress markers (T-AOC, GSH-PX, SOD, MDA, GSH), inflammatory chemokine levels using a Luminex panel, and myocardial SSAO activity via HPLC. The results demonstrated that hydralazine at doses of 5 and 10 mg/kg significantly improved 7-day survival rates from 20% to 90% and enhanced cardiac function in septic mice. It also reduced myocardial injury and histological damage while attenuating systemic inflammation through suppression of chemokine elevation. Furthermore, hydralazine boosted systemic and myocardial antioxidant capacity and normalized the sepsis-induced increase in myocardial SSAO activity, suggesting a potential mechanism for its protective effects. In conclusion, hydralazine shows robust cardioprotection in experimental sepsis by decreasing oxidative stress and inflammatory cell infiltration. The inhibition of SSAO activity may be a pivotal underlying molecular mechanism.

## 1. Introduction

Sepsis was defined as life-threatening organ dysfunction caused by a dysregulated host response to infection in 2016 [1]. Despite significant improvement in understanding and treatment approach to sepsis, it remains a severe health problem worldwide and the major cause of death among critically ill patients in intensive care units (ICUs).

Sepsis-induced myocardial dysfunction (SIMD) is a common complication in septic patients. Approximately 50% of patients with sepsis exhibit impairment of cardiac function [2,3]. SIMD was considered to be characterized by intrinsic myocardial systolic and diastolic dysfunction of the heart [4,5]. Given the central role of the circulatory system in maintaining multiple organs’ functions, SIMD may lead to further deterioration of other organs. It has been reported that the mortality of septic patients with concomitant SIMD is as high as 70–90% [6].

Currently, early effective fluid resuscitation (early goal-directed therapy, EGDT) and dobutamine are the widely accepted practices for SIMD treatment in clinical practice [7,8], EGDT therapy is believed to improve hypoperfusion, while dobutamine increases myocardial contractility. However, there are studies demonstrating that EGDT therapy and dobutamine do not significantly decrease the mortality of SIMD [9]. The high mortality of the SIMD is attributed to intrinsic myocardial systolic and diastolic dysfunction of the heart. This dysfunction is primarily driven by inflammatory and oxidative injury of myocardium [10,11]. However, current clinical management, such as fluid resuscitation and inotropic support, does not directly target these underlying pathogenic processes. Therefore, a strategy targeting myocardial protection might be beneficial for SIMD patients.

Notably, recent studies have highlighted the potential role of semicarbazide-sensitive amine oxidase (SSAO), also known as vascular adhesion protein-1 (VAP-1), in the pathogenesis of sepsis-related organ injury [12]. SSAO is a member of the copper-containing amine oxidase family that exists in both membrane-bound and soluble forms [13]. It catalyzes the deamination of primary amines, producing aldehydes, hydrogen peroxide, and ammonia, which collectively contribute to oxidative stress and inflammatory responses. Elevated SSAO activity has been implicated in leukocyte recruitment and tissue damage in various inflammatory and cardiovascular diseases [14,15]. In the context of sepsis, SSAO may serve as a critical mediator linking inflammatory infiltration to myocardial dysfunction, though its specific role in SIMD remains underexplored.

Hydralazine (HYD) was approved as an anti-hypertensive drug in 1953 by the United States Food & Drug Administration (FDA). Beyond its hemodynamic effects, hydralazine has been demonstrated to exhibit direct cellular protective properties through multifaceted mechanisms. It exhibits potent anti-inflammatory and antioxidant properties [16], modulates apoptosis-related pathways (Bcl-2 family and caspases), and acts as a DNA methylation inhibitor by suppressing DNMT1 and DNMT3a [17,18]. Clinically, hydralazine improves survival and quality of life in African Americans with advanced heart failure, and in combination with isosorbide-dinitrate, alleviates symptoms in heart failure with reduced ejection fraction without causing excessive hypotension [19,20]. Previous studies indicated that hydralazine could reduce myocardial infarct size by decreasing leukocyte infiltration and oxidative stress in a rat model of myocardial ischemia–reperfusion (I/R) injury [21]. Interestingly, beyond its antioxidant and anti-inflammatory properties, hydralazine has been identified as a potent inhibitor of SSAO activity [21]. This additional mechanism may contribute to its cardioprotective effects, particularly in conditions involving oxidative stress and inflammation, such as SIMD.

Many studies have shown that cytokine cascade, inflammatory response, oxidative stress, mitochondrial dysfunction, abnormal energy metabolism, and cell apoptosis are the potential pathogenesis mechanisms of SIMD. Since hydralazine has been shown to possess anti-oxidative effects, and given its inhibitory effect on SSAO, it is rational to hypothesize that hydralazine may exert a therapeutic effect in SIMD. Although prior studies have evaluated hydralazine in short-term sepsis models, such as a 6 h LPS-induced multiorgan dysfunction model [22] and a cecal ligation and puncture model observed for up to 48 h [23], its long-term efficacy and precise cardioprotective mechanisms remain incompletely defined. Therefore, this study aimed to investigate whether hydralazine improves survival and cardiac function over a 7-day period in a murine LPS-induced sepsis model, with a specific focus on the role of SSAO inhibition in its protective mechanism.

## 2. Materials and Methods

### 2.1. Reagents

Lipopolysaccharide (LPS; Escherichia coli O55:B5, L2880) and the monoamine oxidase inhibitors clorgyline (M3778) and pargyline (P8013) were purchased from Sigma-Aldrich (St. Louis, MO, USA). Hydralazine hydrochloride (HYD; H129281) was obtained from Aladdin Bio-Chem Technology Co., Ltd. (Shanghai, China). Isoflurane (R510-22-10) was sourced from RWD Life Science Co., Ltd. (Shenzhen, China). The Bio-Plex Pro Mouse Chemokine Panel (Lot No. 12009159) was from Bio-Rad Laboratories (Hercules, CA, USA). Assay kits for glutathione peroxidase (GSH-PX, A005) and total antioxidant capacity (T-AOC, A015) were purchased from Nanjing Jiancheng Bioengineering Institute (Nanjing, China). The Superoxide Dismutase (SOD, S0101S) and BCA Protein Assay (P0012) kits were obtained from Beyotime Biotechnology (Shanghai, China).

### 2.2. Animal

Male C57BL/6J mice (8–12 weeks old) were purchased from Hunan SJA Laboratory Animal Co., Ltd. (Changsha, China). All animal procedures were approved by the Laboratory Animal Ethics Committee of Shantou University Medical College (approval no. SUMC2022-489; 7 July 2022) and adhered to the National Institutes of Health Guide for the Care and Use of Laboratory Animals. The mice were housed in the Specific Pathogen-Free (SPF) facility of the Shantou University Medical College Laboratory Animal Center under standard conditions: a temperature of 25 ± 1 °C, humidity of 65 ± 5%, and a 12 h light/dark cycle, with ad libitum access to food and water. Prior to experiments, mice were allowed to acclimate to the environment for 7 days.

### 2.3. Experimental Protocols

Sepsis-induced myocardial dysfunction (SIMD) was induced by a single intraperitoneal (i.p.) injection of LPS (25 mg/kg). This dose was selected based on previous literature demonstrating its efficacy in inducing an acute phase of septic shock [24,25] and was confirmed in our preliminary experiments to reproducibly elicit significant cardiac dysfunction. Mice were randomly divided into four groups: Control, HYD, LPS and LPS + HYD. LPS and hydralazine were dissolved in sterile 0.9% saline. Mice in the LPS and LPS + HYD groups received an i.p. injection of LPS (25 mg/kg), while mice in the Control and HYD groups received an equal volume of saline as a vehicle control. Thirty minutes later, mice in the HYD and LPS + HYD groups were administered with hydralazine. Mice in the Control and LPS groups received an equal volume of saline. Hydralazine was administered 30 min after LPS injection. This interval was chosen to model a therapeutic intervention initiated after the onset of sepsis, targeting the early hyperinflammatory and oxidative phase, which peaks within the first hour [26,27]. The endpoint for tissue and plasma collection was set at 24 h after LPS injection. This time point was selected because it represents the established peak of LPS-induced myocardial dysfunction in murine models, allowing for the clear assessment of acute cardiac injury and treatment efficacy [22,28,29,30,31]. All animals were euthanized at 24 h post-LPS injection. Euthanasia was performed by CO_2_ inhalation followed by cervical dislocation, in accordance with the American Veterinary Medical Association (AVMA) Guidelines for the Euthanasia of Animals. Confirmation of death was ensured by the absence of a heartbeat and respiration. Blood and cardiac tissues were collected. Plasma was separated and stored at −80 °C for subsequent analyses.

For the longitudinal assessment of cardiac function by echocardiography, a separate naive control group undergoing repeated measurements was not included. This decision was made to adhere to the principles of Reduction and Refinement in animal research. Using the baseline (0 h) measurement of each experimental group as an internal control minimizes the number of animals used and reduces inter-individual variability, providing a more precise assessment of temporal changes. The HYD-only group served as a procedural control to confirm that the administration of hydralazine and the repeated echocardiographic procedures themselves did not significantly affect cardiac function.

### 2.4. Survival Study

To assess the dose-dependent effect of hydralazine on survival in severe sepsis, sixty mice were randomly assigned to six groups (*n* = 10 per group): Control, LPS, and LPS + HYD (1, 5, 10 and 20 mg/kg). All mice received an i.p. injection of LPS (25 mg/kg) or saline (Control group). Thirty minutes later, hydralazine or saline was administered as described in Section 2.3. Mortality was recorded for 7 days. Concurrently, body weight was measured 1 day before LPS injection and daily thereafter. Anal temperature was monitored for 4 consecutive days post-injection using an electronic thermometer for experimental animals (TWJ, Henan Chico-J-X Technology Co., Ltd., Zhengzhou, China).

Based on the survival results, the 10 mg/kg dose was selected for subsequent mechanistic experiments due to its optimal efficacy. To confirm the dose–response relationship for key biochemical endpoints, the 5 mg/kg dose was also included in specific assays as indicated below.

### 2.5. Echocardiography

Cardiac function in each mouse was assessed by transthoracic echocardiography at baseline (0 h) before LPS or saline injection, and subsequently at 2, 6, 12, and 24 h post-injection using a Vevo 2100 high-resolution imaging system (FUJIFILM VisualSonics, Inc., Toronto, ON, Canada). Mice received a single dose of hydralazine (10 mg/kg) or saline 30 min post-LPS, as described in Section 2.3. Mice were anesthetized with 2% isoflurane for induction and maintained at 0.5% during image acquisition. Two-dimensional B-mode and M-mode images of the left ventricular (LV) short-axis were acquired at the level of the papillary muscles. The LV internal diameters at end-diastole (LVIDd) and end-systole (LVIDs) were measured from M-mode tracings. LV end-diastolic volume (LVEDV) and end-systolic volume (LVESV) were calculated using the Teichholz method: LVEDV = 7.0 × LVIDd^3^/(2.4 + LVIDd) and LVESV = 7.0 × LVIDs^3^/(2.4 + LVIDs) [3]. Subsequently, left ventricular stroke volume (LVSV), ejection fraction (EF), and fractional shortening (FS) were calculated as follows: LVSV = LVEDV − LVESV; EF (%) = (LVEDV − LVESV)/LVEDV × 100%; FS (%) = (LVIDd − LVIDs)/LVIDd × 100%. For each mouse, all parameters were averaged over at least three consecutive cardiac cycles.

### 2.6. Blood Pressure Measurement

For long-term hemodynamic monitoring, a separate cohort of mice was used. Blood pressure and heart rate in conscious mice were monitored using a non-invasive CODA tail-cuff system (Kent Scientific, Torrington, CT, USA) [32]. This setup allowed for non-terminal monitoring over an extended period to assess the time course of hemodynamic recovery. To ensure reliable measurements, mice were trained for 14 consecutive days prior to data collection. Baseline measurements were recorded 24 h before LPS injection. Subsequent measurements were taken at 2, 6, 12, 24, 48, 72, and 96 h post-LPS administration for all experimental groups.

### 2.7. Blood Plasma Biochemical Analyses

Blood plasma levels of myocardial injury markers, including creatine kinase (CK), creatine kinase-MB (CK-MB), lactate dehydrogenase (LDH), and aspartate aminotransferase (AST), were measured in the Control, LPS, and LPS + HYD (5 and 10 mg/kg) groups, were measured using an automatic biochemical analyzer (ADVIA^®^ 2400, Siemens Healthcare, Erlangen, Germany).

### 2.8. Histological Evaluation

#### 2.8.1. Hematoxylin-Eosin (H&E) Staining

Cardiac tissues from the Control, LPS, and LPS + HYD (10 mg/kg) groups were subjected to histopathological evaluation. Following blood collection, mouse hearts were rapidly excised and fixed in 4% paraformaldehyde for at least 24 h. The left ventricles were then embedded in paraffin and sectioned at a thickness of 3–4 μm. Sections were stained with Hematoxylin and Eosin (H&E) to evaluate histopathological changes, including inflammatory cell infiltration, erythrocyte extravasation, and alterations in myocardial structure. Digital images were acquired using a slide scanner (iScan Coreo, Roche, Mannheim, Germany) and analyzed with VENTANA Image Viewer software (version 3.1.4) (Ventana Medical Systems, Inc., Marana, AZ, USA). The extent of myocardial injury was assessed using a semi-quantitative histological approach. From each heart, five separate sections were randomly selected and examined under a light microscope. Tissue damage was graded on a five-point severity scale according to the method established by Hadi et al. (Table 1) [33]. To minimize observer bias, all histological evaluations were performed independently by two pathologists blinded to group assignments.

#### 2.8.2. Transmission Electron Microscopy (TEM)

Cardiac tissues from the Control, LPS, and LPS + HYD (10 mg/kg) groups were used for ultrastructural evaluation. Following euthanasia, mouse hearts were perfused with cold saline and dissected into 1 mm^3^ cubes. The tissue cubes were fixed in 2.5% glutaraldehyde for at least 3 h, followed by post-fixation in 1% osmium tetroxide for 1 h. Subsequently, samples were dehydrated through a graded ethanol series, stained with 2% uranyl acetate, and embedded in resin overnight. Ultrathin sections (approximately 70 nm) were collected on copper grids and examined using a JEM1400 transmission electron microscope (JEOL Ltd., Tokyo, Japan) operating at an accelerating voltage of 80 kV.

For semi-quantitative analysis, a mitochondrial injury scoring system was adapted [34]. For each mouse, three non-overlapping ultrathin sections were randomly selected, and 10 random fields per section were captured at a standardized magnification (30,000×). Mitochondrial damage in each field was graded on a six-point scale (Table 1). The final injury score for each mouse was the average of all fields analyzed. To ensure objectivity and minimize observer bias, all images were scored by two independent investigators who were blinded to the group assignments. Any discrepancies in scoring were resolved through consensus.

### 2.9. Quantification of Blood Plasma Chemokines

Plasma samples from the Control, LPS, and LPS + HYD (10 mg/kg) groups were analyzed. The concentrations of 15 chemokines in plasma were quantified using the Bio-Plex Pro Mouse Chemokine Panel (Bio-Rad Laboratories, Hercules, CA, USA) according to the manufacturer’s instructions. The 15 chemokines assessed in this study were: CCL1, CCL2, CCL3, CCL4, CCL5, CCL7, CCL11, CCL12, CCL17, CCL20, CCL22, CCL24, CXCL1, CXCL10, and CXCL16. Samples were diluted 1:4 and measured in duplicate on a Luminex 200 system (Bio-Rad Laboratories, Inc., Hercules, CA, USA). Data acquisition and analysis were performed using Milliplex Analyst software (version 5.1).

### 2.10. Plasma GSH-PX, T-AOC, SOD Activity Measurement

Plasma samples from the Control, LPS, and LPS + HYD (5 and 10 mg/kg) groups were analyzed. The activities of glutathione peroxidase (GSH-PX), total antioxidant capacity (T-AOC), and superoxide dismutase (SOD) were measured using commercial assay kits. Specifically, GSH-PX activity was determined by a colorimetric assay, T-AOC by an ABTS (2,2′-azino-bis(3-ethylbenzthiazoline-6-sulfonic acid))-based assay, and SOD activity by a WST-8 assay, all performed according to the respective manufacturer’s protocols [35,36,37].

### 2.11. Determination of Malondialdehyde (MDA) and Glutathione (GSH) in Plasma and Cardiac Tissue

Plasma and cardiac tissue samples from the Control, LPS, and LPS + HYD (5 and 10 mg/kg) groups were analyzed. Cardiac tissues were excised, rinsed with ice-cold saline, blotted dry, and weighed. After mincing in ice-cold PBS (0.1 M, pH 7.4), the tissues were washed three times by centrifugation (1000 g, 2 min, 4 °C). The resulting pellets were then homogenized (10% *w*/*v*) in PBS on ice by using an IKA homogenizer and centrifuged at 12,000× *g* for 15 min at 4 °C. The final supernatants were collected for subsequent assays.

MDA concentration was determined by High-Performance Liquid Chromatography (HPLC) with a 2-thiobarbituric acid (TBA) reaction, as previously described with minor modifications [38]. Briefly, aliquots (50 μL) of serum or cardiac tissue homogenates were mixed with 100 μL of 0.3% orthophosphoric acid and 100 μL of 0.6% TBA. The mixture was vortexed, heated at 90 °C for 30 min, and then cooled on ice. After extraction with 250 μL of n-butanol and phase separation facilitated by 50 μL of saturated NaCl, samples were centrifuged at 12,000× *g* for 1 min. The upper butanol phase (150 μL) was collected for HPLC analysis. Chromatographic separation was performed on an analytical column (150 × 4.6 mm, 5 μm) equipped with a guard column (4.0 × 4.0 mm, 5 μm), using a mobile phase of 50 mmol/L ammonium acetate and methanol (60:40, *v*/*v*) at a flow rate of 1.0 mL/min. Fluorescence detection was set at excitation (λex) and emission (λem) wavelengths of 527 nm and 551 nm, respectively. A standard curve was generated using known concentrations of 1,1,3,3-tetramethoxypropane (TMP), which is a stable MDA precursor, to ensure accurate quantification.

GSH concentration was measured by HPLC following derivatization with 2-chloro-1-methylquinolinium tetrafluoroborate (CMQT), according to established protocols [39,40]. To summarize, all samples were reduced with tris (2-carboxyethyl) phosphine, derivatized with CMQT, and separated on a Zorbax SB-C18 column (4.6 × 150 mm, 5 μm, Agilent) and detected with UV detection at 355 nm.

The concentrations of MDA and GSH in cardiac tissues were normalized to the total protein content of each sample.

### 2.12. Measurement of Myocardial SSAO Activity

Cardiac tissue samples from the Control, LPS, and LPS + HYD (5 and 10 mg/kg) groups were analyzed. Myocardial semicarbazide-sensitive amine oxidase (SSAO) activity was determined by HPLC using benzylamine as the substrate, as previously described [21,41]. Cardiac tissues were homogenized (10% *w*/*v*) in ice-cold 0.1 M PBS (pH 7.4), using an IKA homogenizer on ice, following the same procedure described in Section 2.11. Then, 200 μL of tissue homogenate was pre-incubated for 30 min at room temperature with the monoamine oxidase-A (MAO-A) inhibitor clorgyline (10 μmol/L) and the monoamine oxidase-B (MAO-B) inhibitor pargyline (10 μmol/L) to ensure specific measurement of SSAO. Following the addition of benzylamine, the reaction mixture was incubated at 37 °C for 1 h. The amount of benzaldehyde produced was then quantified by HPLC with fluorometric detection. Total protein concentration in the heart tissues was measured using a BCA protein assay kit. SSAO activity was expressed as nanomoles of benzaldehyde generated per hour per milligram of protein (nmol/h/mg protein).

### 2.13. Statistical Analysis

All statistical analyses were performed using GraphPad Prism software (version 8.2, GraphPad Software, La Jolla, CA, USA). Data are presented as mean ± standard deviation (SD). The normality of data distribution was assessed using the Kolmogorov–Smirnov test. For comparisons among multiple groups, a one-way analysis of variance (ANOVA) was conducted, followed by Tukey’s post hoc test for pairwise comparisons. For data that were not normally distributed, the non-parametric Kruskal–Wallis test was applied. Survival rates were analyzed using the Kaplan–Meier method. A *p*-value of less than 0.05 (*p* < 0.05) was considered statistically significant.

## 3. Results

### 3.1. Hydralazine Improved the Survival Rates of Septic Mice Induced by LPS

**Figure 1:**
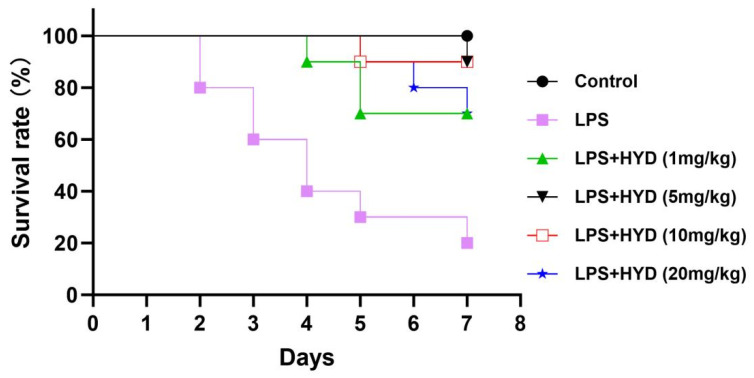
Kaplan–Meier survival curves for LPS-induced septic mice treated with varying doses of hydralazine. Mice were monitored for 14 days, and all mortality occurred within the first 7 days (*n* = 10 per group).

As shown in Figure 1, all mice in control group (saline) survived during the entire observation period (7 days). Mice in LPS group had a survival rate of 20% after 7 days’ observation. Seven days after sepsis induction, the LPS + HYD (5 mg/kg) and LPS + HYD (10 mg/kg) groups showed only one death in each group (90% survival rate). The mice survival rates in LPS + HYD (1 mg/kg) and LPS + HYD (20 mg/kg) groups were 70%. Based on result of the survival study, we chose 10 mg/kg as the dose of hydralazine for subsequent mechanistic studies to investigate its effects on cardiac dysfunction and key pathological pathways in an acute septic shock model.

The physiological decline in septic mice was further assessed by monitoring body weight and anal temperature. Mice in the control group maintained stable body weight and temperature throughout the observation period. In contrast, LPS administration induced a significant reduction in both body weight and core body temperature (Supplementary Figure S1). Body weight loss reached its nadir on day 4 post-LPS and remained at this low level, while the lowest temperature was observed during the first two days, followed by a slight recovery. Treatment with hydralazine (1–20 mg/kg) significantly attenuated the LPS-induced hypothermia within 2 days and ameliorated the body weight loss by day 4, in a dose-dependent manner. These data indicate that hydralazine improves the overall physiological condition of septic mice, corroborating its survival benefit.

### 3.2. Characterization of the LPS-Induced Sepsis Model and Its Impact on Cardiac Function and Hemodynamics

**Figure 2:**
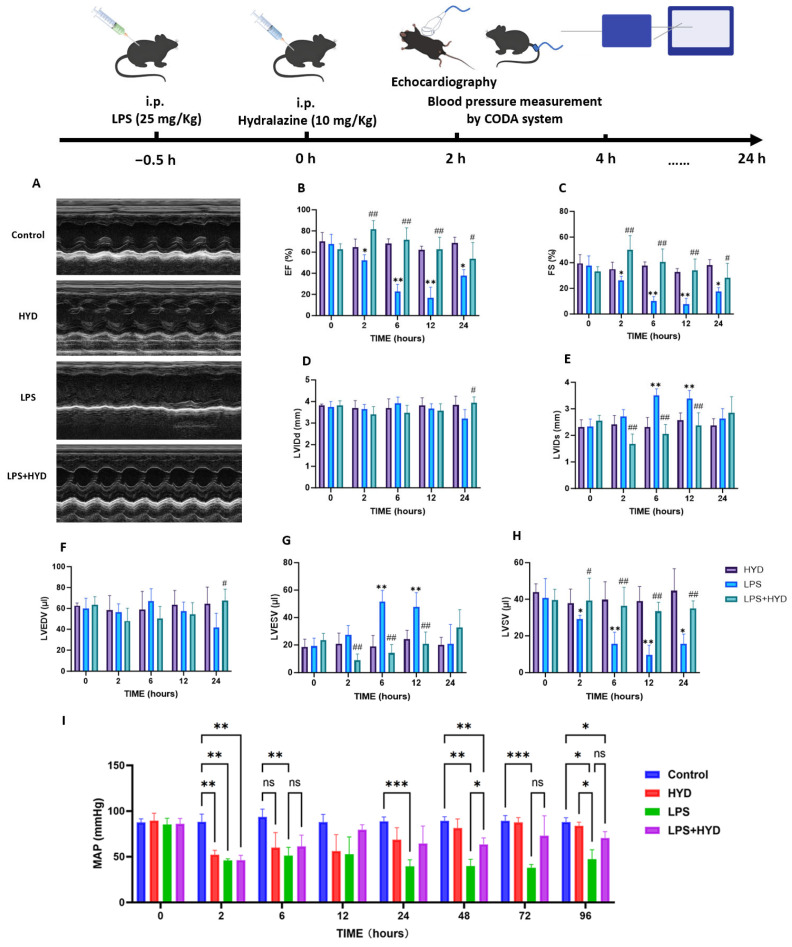
Effect of hydralazine on cardiac function and blood pressure in mice with SIMD during the acute septic shock phase. (**A**). Representative M-mode echocardiograms. The image labeled ‘Control’ shows the baseline (0 h) recording from the LPS group, which was representative of all groups prior to treatment. The other group images were recorded at the 12 h time point (*n* = 5 per group). Representative M-mode echocardiograms for all experimental groups across key time points (baseline, 2, 6, 12, 24 h post-LPS) are provided in Supplementary Figure S2. (**B**–**H**). Left ventricular ejection fraction (EF%), left ventricular fraction shortening (FS%), left ventricular internal diameters at end-diastole (LVIDd), left ventricular internal diameters at end-systole (LVIDs), left ventricular end-diastolic volume (LVEDV), left ventricular end-systolic volume (LVESV) and left ventricular stroke volume (LVSV) were quantified via echocardiography (*n* = 5 for each group). Data are expressed as mean ± SD. * *p* < 0.05 and ** *p* < 0.01 vs. the baseline, # *p* < 0.05 and ## *p* < 0.01 vs. the LPS group at the corresponding time point. (**I**) Mean arterial pressure (MAP) was quantified via the CODA system (*n* = 4 for Control, HYD and LPS + HYD groups, *n* = 8 for LPS group). Four mice died during the experiment in the LPS group. Data are expressed as mean ± SD. * *p* < 0.05, ** *p* < 0.01 and *** *p* < 0.001 vs. corresponding group.

To characterize the septic model, we first assessed the effects of LPS administration on cardiac function, hemodynamics, and myocardial injury. Echocardiography revealed that LPS induced a rapid and severe deterioration in cardiac function (Figure 2A–H). Compared with the baseline, the cardiac function was apparently impaired in LPS group. EF% and FS% of mice at 12 h time point were significantly lower than those of baseline in LPS group (EF%: 16.85 ± 9.91% vs. 67.78 ± 9.10%, *p* < 0.01; FS%: 7.51 ± 4.70% vs. 37.56 ± 7.74%, *p* < 0.01). LVESV of mice at 6 h and 12 h time points were significantly higher than those of baseline in LPS group (6 h: 51.72 ± 8.09 μL, 12 h: 47.76 ± 10.42 μL vs. baseline: 19.08 ± 5.85 μL *p* < 0.01). LVSV of mice at 12 h time point was significantly decreased than those of baseline in LPS group (9.51 ± 5.36 μL vs. 40.85 ± 10.46 μL, *p* < 0.01). However, there was no difference in LVEDV (57.27 ± 8.85 μL, 67.23 ± 11.62 μL vs. 59.93 ± 9.88 μL, *p* > 0.05) among these three groups. The stability of LVEDV, despite the vasodilatory properties of LPS and hydralazine, may reflect the complex interplay between reduced preload and impaired contractility that characterizes septic cardiomyopathy.

Concurrently, LPS administration induced profound and sustained hypotension (Figure 2I), with MAP decreasing from a baseline of 85.25 ± 6.99 mmHg to 46.00 ± 1.82 mmHg at 2 h (*p* < 0.001) and failing to recover within the 96 h observation period.

**Figure 3:**
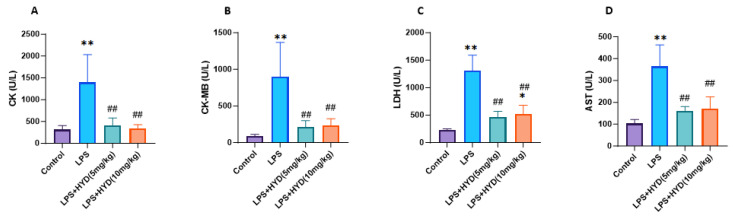
Effects of LPS-induced sepsis and hydralazine on myocardial injury markers (CK, CK-MB, LDH and AST) levels in the blood plasma (*n* = 6 for each group). Mice were randomly divided into four groups (LPS, LPS + HYD 5 mg/kg, LPS + HYD 10 mg/kg and Control). LPS + HYD and LPS groups were administered LPS at a dose of 25 mg/kg via i.p. injection, after 30 min received hydralazine (5 mg/kg or 10 mg/kg) or equal saline, respectively. Control group was administered equal saline. The measurements were taken at 24 h post-LPS injection, during the acute phase of septic shock. (**A**). CK, creatine kinase; (**B**). CK-MB, creatine kinase MB; (**C**). LDH, lactate dehydrogenase (LDH); (**D**). AST aspartate aminotransferase. * *p* < 0.05 and ** *p* < 0.01 vs. the control group, ## *p* < 0.01 vs. the LPS group.

This functional impairment induced by LPS was accompanied by significant myocardial injury, as evidenced by the dramatic elevation of plasma markers including CK (1395 ± 637.7 U/L), CK-MB (895 ± 472.5 U/L), LDH (1309 ± 282 U/L) and AST (364.2 ± 98.96 U/L) after 24 h of LPS injection, compared to the control group (all *p* < 0.01) (Figure 3).

**Figure 4:**
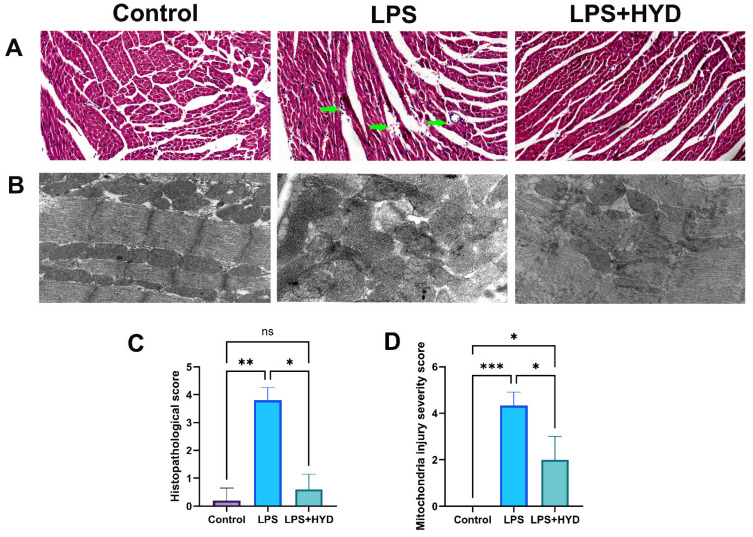
Photomicrographs of myocardial tissue in septic mice. Representative images and quantitative analysis of myocardial tissue were obtained from each experimental group during the acute septic shock phase (24 h post-LPS). (**A**). Representative HE staining photomicrographs (3 μm, magnification ×200, *n* = 5 samples/mice, 5 mice/group). The LPS group exhibited leukocyte infiltration and increased intramyocardial hemorrhage (green arrow), while the LPS + HYD group showed significantly less leukocyte infiltration and intracardiac hemorrhage. (**B**). Representative TEM ultrastructure images of myocardial tissue sections from each experimental group (magnification ×30,000, *n* = 3). (**C**). Semi-quantitative analysis of histopathological injury based on H&E staining. (**D**). Semi-quantitative analysis of mitochondrial damage based on TEM images. * *p* < 0.05, ** *p* < 0.01 and *** *p* < 0.001.

Histological and ultrastructural examinations confirmed extensive myocardial damage [42]. H&E staining revealed that the control group had a normal myocardial structure with an injury score of 0.2 ± 0.44. In contrast, LPS group mice exhibited severe pathological changes, including disordered myocardial fibers, edema, and extensive inflammatory cell infiltration, resulting in a significantly higher injury score (3.8 ± 0.45, *p* < 0.001 vs. Control). (Figure 4A,C). Consistent with these findings, TEM analysis of ultrastructural changes further confirmed the LPS-induced myocardial injury. The control group displayed well-organized myofibrils and intact mitochondria with a mitochondrial injury score of 0 (Figure 4B,C). The LPS group showed severe mitochondrial damage, characterized by swelling, cristae disruption, and fragmentation, leading to a significantly increased injury score (4.33 ± 0.57, *p* < 0.001 vs. Control). 

### 3.3. Hydralazine Improved Cardiac Function and Hemodynamics in Mice with SIMD

Having established the severity of LPS-induced cardiac dysfunction, we next evaluated the therapeutic potential of hydralazine administered during the early septic phase (30 min post-LPS). Cardiac function and hemodynamics were serially monitored after LPS challenge, with hydralazine administered 30 min post-LPS.

At the 2 h time point, coinciding with the peak of the initial inflammatory and hypotensive response, a significant drop in mean arterial pressure (MAP) was observed in both the LPS (46.00 ± 1.82 mmHg) and LPS + HYD (46.25 ± 5.25 mmHg) groups compared to their baselines and the Control group (*p* < 0.001; Figure 2I). This indicates that hydralazine did not exacerbate the early septic hypotension. Echocardiography at this early stage revealed the beginning of cardiac contractile impairment in the LPS group, as evidenced by a significantly decreased EF%, ES%, and LVSV (Figure 2B,C,H). The LPS + HYD group began to show a protective trend, with EF%, ES%, and LVSV values higher than those in the LPS group, while, LVESV values lower than those in the LPS group (*p* < 0.01) (Figure 2B–G). The HYD-alone group showed a transient decrease in MAP at 2 h but normal cardiac function, confirming the drug’s known vasodilatory effect without direct cardiotoxicity.

By 6 h post-LPS, cardiac dysfunction in the LPS group became more pronounced. LVESV was significantly elevated (51.72 ± 8.09 μL, *p* < 0.01 vs. baseline), and EF%, FS%, and LVSV showed significant declines (Figure 2B,C,G,H). This demonstrates the progressive nature of septic cardiomyopathy. The LPS + HYD group shows a protective effect, with EF%, ES%, and LVSV values higher than those in the LPS group, while, LVESV values lower than those in the LPS group (*p* < 0.01) (Figure 2G). MAP remained severely depressed in the LPS group, while the LPS + HYD group’s MAP was comparable, and the HYD group’s pressure had normalized.

The most severe cardiac impairment was observed at 12 h. The LPS group reached a nadir in contractility, with EF% and FS% plummeting to 16.85 ± 9.91% and 7.51 ± 4.70%, respectively (*p* < 0.01 vs. baseline; Figure 2B,C). Stroke volume (LVSV) was critically reduced to 9.51 ± 5.36 μL (*p* < 0.01 vs. baseline). This confirms 12 h as the peak window of LPS-induced myocardial stunning in this model. In contrast, hydralazine treatment conferred remarkable protection at this time point. The LPS + HYD group exhibited significantly higher EF% (62.85 ± 11.28%, *p* < 0.01) and FS% (33.92 ± 9.06%, *p* < 0.01), and markedly improved LVESV (20.86 ± 8.59 μL, *p* < 0.01) and LVSV (33.39 ± 4.90 μL, *p* < 0.01) compared to the LPS group. These data unequivocally demonstrate that hydralazine effectively preserves cardiac contractile function during the acute phase of sepsis.

At 24 h, the LPS group showed no signs of functional recovery. Conversely, the LPS + HYD group maintained significantly better cardiac function, with EF% and FS% remaining at near-normal levels, underscoring the sustained therapeutic effect of hydralazine. Hemodynamically, a key divergence emerged at later time points. While the LPS group remained profoundly hypotensive, the LPS + HYD group showed a significant acceleration in recovery, with MAP at 48 h being significantly higher than in the LPS group (63.5 ± 7.23 mmHg vs. 39.66 ± 7.57 mmHg, *p* < 0.05; Figure 2I). This suggests that hydralazine contributes to stabilization of overall cardiovascular homeostasis.

### 3.4. The Protection Effect of Hydralazine Against Tissue Damage Induced by Sepsis

We then investigated whether hydralazine could attenuate the myocardial injury identified in the model. The administration of hydralazine (5 and 10 mg/kg) significantly reduced sepsis-induced myocardial injury in mice as reflected by the decreased release of CK (5 mg/kg: 413.3 ± 169.3 U/L; 10 mg/kg: 344 ± 83.87 U/L), CK-MB (5 mg/kg: 211.5 ± 89.69 U/L; 10 mg/kg: 234.3 ± 91.62 U/L), LDH (5 mg/kg: 463.7 ± 107.7 U/L; 10 mg/kg: 521.4 ± 160.9 U/L) and AST (5 mg/kg: 161.3 ± 19.28 U/L; 10 mg/kg: 170.5 ± 54.09 U/L) (Figure 3) (*p* < 0.01), indicating hydralazine’s protective effects against LPS induced myocardial damage in vivo.

Furthermore, hydralazine (10 mg/kg) ameliorated the histopathological and ultrastructural damage. In the LPS + HYD group, the arrangement of myocardial fibers was relatively ordered, and myocardial edema, haemorrhage, necrosis, and inflammatory cell infiltration were apparently improved compared to the LPS group (Figure 4A). The semi-quantitative analysis of the H&E-stained sections showed a significant increase in histopathological scores in the LPS group compared to the Control group (*p* < 0.01), which was markedly attenuated by hydralazine treatment (*p* < 0.05) (Figure 4C).

Representative transmission electron microscopy (TEM) images provided ultrastructural evidence. Compared to the LPS group, myocardial tissues from the LPS + HYD group exhibited an apparent improvement in morphology, including reduced myofibrillar disarray and mitochondrial swelling. The architecture of mitochondrial cristae also appeared more distinct and better preserved. The results of semi-quantitative analysis of mitochondrial ultrastructure showed a significant increase in mitochondrial damage score in the LPS group compared to the Control group (*p* < 0.001), which was significantly attenuated by hydralazine treatment (*p* < 0.05) (Figure 4D). These results indicated that hydralazine could attenuate LPS-induced ultrastructural damage in cardiomyocytes.

### 3.5. Hydralazine Suppressed the Systemic Inflammatory Response in Sepsis

**Figure 5:**
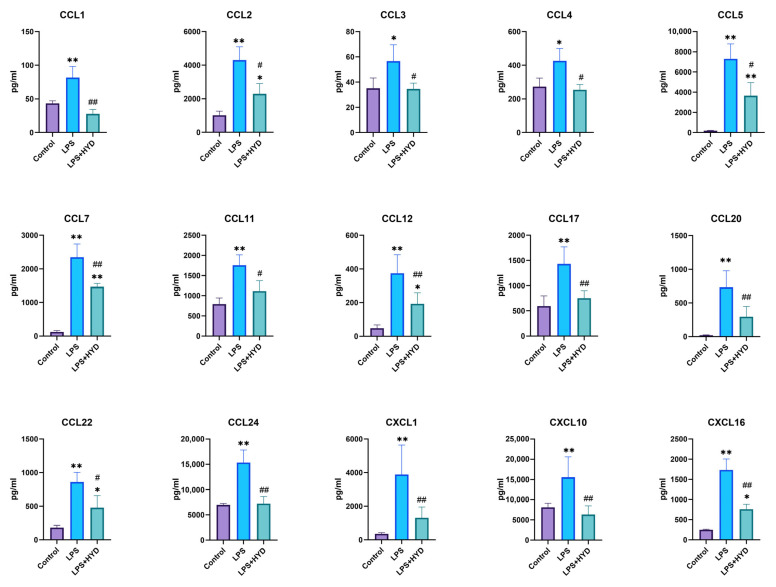
Changes of 15 blood plasma cytokines in septic mice (*n* = 5 for each group). Mice were randomly divided into three groups (LPS, LPS + HYD 10 mg/kg and Control). LPS + HYD and LPS groups were administered LPS at a dose of 25 mg/kg via i.p. injection, after 30 min received hydralazine (10 mg/kg) or equal saline via i.p. injection, respectively. Control group was administered equal saline. Blood plasma was collected at 24 h post-LPS injection (the acute phase of septic shock) to detect chemokines levels with Luminex assay. * *p* < 0.05 and ** *p* < 0.01 vs. the control group, # *p* < 0.05 and ## *p* < 0.01 vs. the LPS group. CCL, C-C motif chemokine ligand; CXCL, C-X-C motif chemokine ligand.

To investigate the effect of hydralazine on the inflammatory response, we quantified a panel of 15 chemokines that are known to be mediators of leukocyte trafficking [43]. These chemokines were measured using a commercially available multiplex assay. As shown in Figure 5, the levels of these chemokines, including CCL1, CCL2, CCL3, CCL4, CCL5, CCL7, CCL11, CCL12, CCL17, CCL20, CCL22, CCL24, CXCL1, CXCL10, and CXCL16 were significantly elevated in the sepsis group compared to the control group (*p* < 0.05 or *p* < 0.01). Following treatment with hydralazine, these elevated chemokine levels induced by LPS were significantly decreased (*p* < 0.05 or *p* < 0.01).

### 3.6. Hydralazine Enhanced Antioxidant Capacity and Attenuated Oxidative Stress

**Figure 6:**
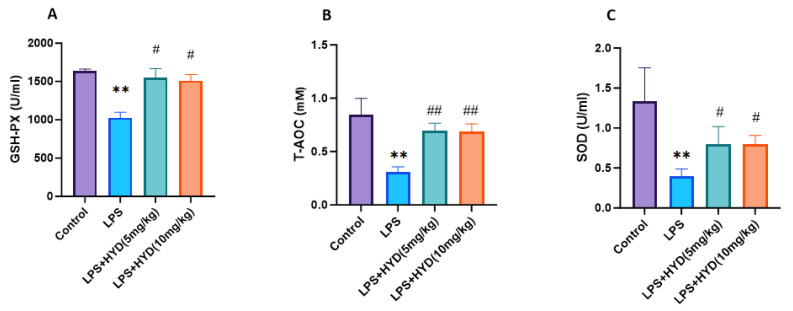
Changes in blood plasma GSH-PX, T-AOC, and SOD activities during the acute phase of sepsis (24 h post-LPS injection) (*n* = 5 for each group). Mice were randomly divided into four groups (LPS, LPS + HYD 5 mg/kg, LPS + HYD 10 mg/kg and Control). LPS + HYD and LPS groups were administered LPS at a dose of 25 mg/kg via i.p. injection, after 30 min received hydralazine (5 mg/kg or 10 mg/kg) or equal saline solution, respectively. Control group was administered equal saline. ** *p* < 0.01 vs. the control group, # *p* < 0.05 and ## *p* < 0.01 vs. the LPS group. (**A**). GSH-PX, glutathione peroxidase; (**B**). T-AOC, the total antioxidant capacity; (**C**). SOD, superoxide dismutase.

As shown in Figure 6, plasma GSH-PX (1026 ± 71.83 U/mL), T-AOC (0.31 ± 0.04 mM) and SOD (0.39 ± 0.09 U/mL) were significantly lower in the LPS group than those in the control group (GSH-PX: 1634 ± 28.25 U/mL, T-AOC: 0.85 ± 0.15 mM and SOD: 1.38 ± 0.41 U/mL, respectively) (*p* < 0.01). After administration of different concentration hydralazine, plasma GSH-PX (5 mg/kg: 1552 ± 114.8 U/mL, 10 mg/kg: 1513 ± 77.37 U/mL), T-AOC (5 mg/kg: 0.70 ± 0.06 mM, 10 mg/kg: 0.69 ± 0.07 mM) and SOD (5 mg/kg: 0.80 ± 0.22 U/mL, 10 mg/kg: 0.80 ± 0.11 U/mL) significantly increased, compared with those in LPS group (*p* < 0.05), but were not significantly different compared with the control group.

**Figure 7:**
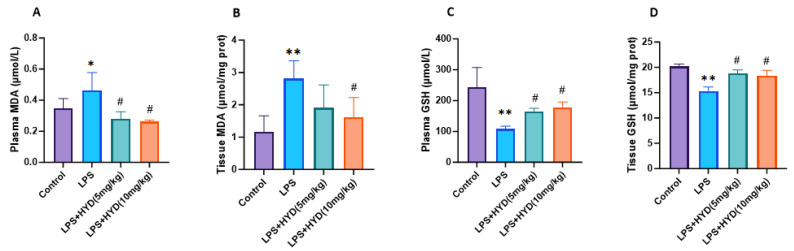
Detection of MDA and GSH concentration in plasma and myocardial tissue by high-performance liquid chromatography in various experimental groups during the acute phase (*n* = 6 for each group). Mice were randomly divided into four groups (LPS, LPS + HYD 5 mg/kg, LPS + HYD 10 mg/kg and Control). LPS + HYD and LPS groups were administered LPS at a dose of 25 mg/kg via i.p. injection, after 30 min received hydralazine (5 mg/kg or 10 mg/kg) or equal saline solution, respectively. Control group was administered equal saline. * *p* < 0.05 and ** *p* < 0.01 vs. Control group, # *p* < 0.05 vs. LPS group. (**A**). blood plasma MDA; (**B**). cardiac tissue MDA; (**C**). blood plasma GSH; (**D**). cardiac tissue GSH. MDA, malondialdehyde. GSH, glutathione.

The level of plasma MDA (0.46 ± 0.11 μmol/L) in the LPS group was significantly higher than the control group (0.34 ± 0.06 μmol/L) (*p* < 0.05). Compared to the LPS group, MDA levels were significantly lower in the LPS + HYD (5 mg/kg) group (0.28 ± 0.04 μmol/L) and the LPS + HYD (10 mg/kg) group (0.26 ± 0.01 μmol/L) (*p* < 0.05) (Figure 7A).

The cardiac tissue MDA level in the LPS group (2.82 ± 0.50 μmol/mg protein) was significantly higher than that in the control group (1.17 ± 0.49 μmol/mg protein) (*p* < 0.05). Compared to the LPS group, treatment with hydralazine reduced MDA levels; this reduction was not statistically significant at the 5 mg/kg dose (1.90 ± 0.70 μmol/mg protein, *p* > 0.05) but was significant at the 10 mg/kg dose (1.62 ± 0.40 μmol/mg protein, *p* < 0.05) (Figure 7B).

The plasma GSH level (107.50 ± 9.79 μmol/L) of the LPS group was significantly decreased compared to the control group (243.20 ± 64.17 μmol/L) (*p* < 0.01). Compared to the LPS group, GSH levels were significantly higher in the LPS + HYD (5 mg/kg) group (164.80 ± 10.48 μmol/L) and the LPS + HYD (10 mg/kg) group (178.40 ± 16.70 μmol/L) (*p* < 0.05) (Figure 7C).

The cardiac tissue GSH level (15.29 ± 0.84 μmol/mg protein) of the LPS group was significantly decreased compared to the control group (20.18 ± 0.47 μmol/mg protein). Compared to the LPS group, GSH levels were significantly higher in the LPS + HYD (5 mg/kg) group (18.80 ± 0.70 μmol/mg protein) and the LPS + HYD (10 mg/kg) group (18.34 ± 1.00 μmol/mg protein) (*p* < 0.05) (Figure 7D).

### 3.7. Hydralazine Inhibited Myocardial SSAO Activity

**Figure 8:**
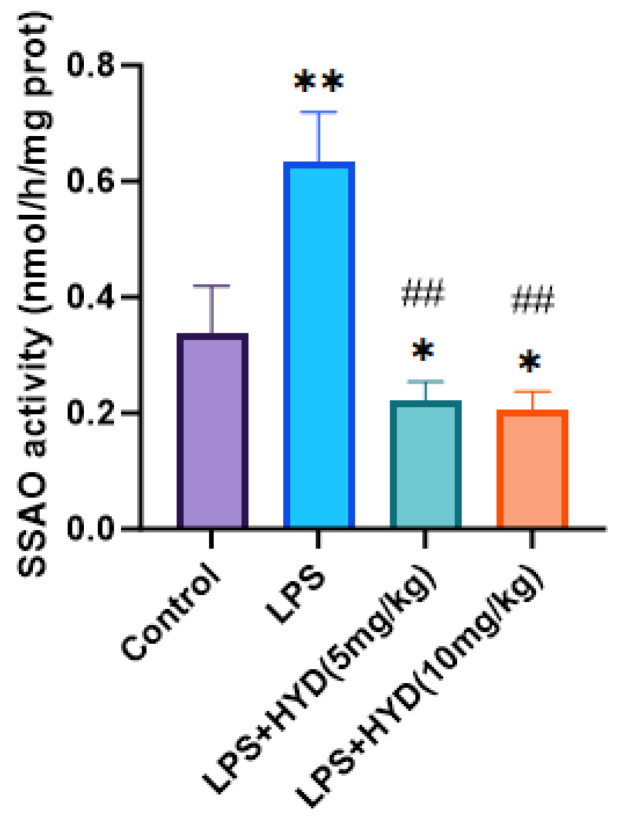
Detection of Myocardial SSAO activity in myocardial tissue by high-performance liquid chromatography in various experimental groups during the acute phase (*n* = 6 for each group). Mice were randomly divided into four groups (LPS, LPS + HYD 5 mg/kg, LPS + HYD 10 mg/kg and Control). LPS + HYD and LPS groups were administered LPS at a dose of 25 mg/kg via i.p. injection, after 30 min received hydralazine (5 mg/kg or 10 mg/kg) or equal saline solution, respectively. Control group was administered equal saline. * *p* < 0.05 and ** *p* < 0.01 vs. Control, ## *p* < 0.01 vs. LPS group.

As shown in Figure 8, HPLC analysis demonstrated that the myocardial SSAO activity was remarkably increased in the LPS group (0.63 ± 0.08 nmol/h/mg protein) compared with the control group (0.34 ± 0.08 nmol/h/mg protein) (*p* < 0.01). Compared to the LPS group, administration of hydralazine significantly decreased SSAO activity in myocardial tissue in both the LPS + HYD (5 mg/kg) (0.22 ± 0.03 nmol/h/mg protein) and LPS + HYD (10 mg/kg) (0.21 ± 0.03 nmol/h/mg protein) groups (*p* < 0.01).

## 4. Discussion

This study demonstrates that hydralazine ameliorates LPS-induced myocardial dysfunction and tissue injury by reducing oxidative stress and inflammatory cell infiltration. This protective effect is likely mediated by the inhibition of SSAO activity, which represents a key underlying mechanism for hydralazine’s cardioprotective effect in septic mice.

Hydralazine, as an old anti-hypertensive drug, induces direct relaxation of arteriolar smooth muscle, without significant effect on capacitance vessels or venous smooth muscle [44]. Beyond its cardiovascular effects (blood pressure control and heart failure therapy), hydralazine exhibits various biological activities, including aldehyde scavenging, demethylation, anti-fibrotic, antitumor, anti-inflammatory, etc. [45,46,47,48,49,50,51]. Notably, hydralazine is a potent irreversible inhibitor of SSAO, with an IC50 in the nanomolar range in rat aorta and heart [52,53]. Our previous research suggested that the mechanism of hydralazine’s anti-tumor effect may also involve SSAO inhibition [54]. SSAOs belong to the copper-containing amine oxidase family and exist in two forms in mammals: a membrane-bound form and a soluble form in plasma. Membrane-bound SSAO is abundant in blood vessels, especially in the smooth muscle layer. SSAO is also expressed in other types of cells, such as endothelial cells, adipocytes and chondrocytes. It catalyzes the oxidative deamination of primary amines, producing aldehydes, ammonia, and hydrogen peroxide, and is implicated in various pathological conditions, including inflammation, obesity, chronic liver disease, cardiovascular disease, stroke, Alzheimer’s disease and diabetes [13].

A study of myocardial ischemia–reperfusion (I/R) injury in Sprague-Dawley rats demonstrated that myocardial SSAO activity was up-regulated, and administration of hydralazine prior to reperfusion significantly improved acute myocardial I/R injury [21]. Sepsis-induced myocardial dysfunction (SIMD) is associated with the prognosis of sepsis, with a mortality rate of over 70%, much higher than in those sepsis patients without cardiac injury (20%), and effective therapeutic options remain lacking [6]. Building upon the protective role of hydralazine in I/R injury and the urgent need for SIMD therapies, this study aimed to evaluate the therapeutic potential and mechanism of hydralazine in a murine model of SIMD.

Unlike studies employing a preventative approach where agents are administered before LPS challenge [55,56], we initiated hydralazine treatment 30 min after sepsis induction for modeling a therapeutic intervention after disease onset, which more accurately reflects the clinical reality where treatment begins after symptom presentation. In addition, considering that some mice with sepsis experienced late-stage death, we extended the observation period of the survival study to 7 days, which was longer than that in many previous studies. This study demonstrated that hydralazine treatment significantly improved survival rates in a mouse model of SIMD.

We further observed the effects of hydralazine on cardiac function and blood pressure in septic mice. The results showed that cardiac contractile function significantly deteriorated as early as 2 h after LPS injection and was most severely impaired at 12 h. Hydralazine administration effectively reversed this dysfunction, restoring myocardial contractility to near-normal levels. Notably, the stability of left ventricular end-diastolic volume (LVEDV) across groups indicates preserved diastolic function, which appears to sharpen the diagnostic focus onto systolic contractility as the primary locus of injury. These findings support the interpretation that the cardioprotective effect of hydralazine may be achieved primarily through the amelioration of systolic failure in our model. The results showed that the administration of hydralazine (10 mg/kg) to healthy control mice induced a transient reduction in blood pressure, with the peak hypotensive effect occurring at 2 h and a gradual return to baseline levels within 24 to 48 h. In LPS-challenged mice already experiencing profound hypotension, the same dose of hydralazine did not exacerbate the blood pressure decline. Instead, it significantly ameliorated the LPS-induced hypotension by 48 h (Figure 2I). This distinct hemodynamic profile strongly suggests that the improved survival and cardiac function conferred by hydralazine are not secondary to its blood pressure-lowering effect, but rather stem from its direct cardiac-protective mechanisms. Collectively, the data presented in Figure 2 capture the profound hemodynamic and functional deterioration characteristic of the acute septic shock response induced by LPS, and demonstrate that hydralazine provides significant stabilization during this critical phase.

Then, we examined the direct evidence of myocardial tissue damage. In agreement with the literature [57], our results showed significant elevation of myocardial injury markers (CK, CK-MB, LDH, AST) in LPS-challenged mice, confirming substantial myocardial tissue damage in SIMD. This biochemical evidence of cellular injury directly corresponds to the observed deterioration in cardiac contractile function. The administration of hydralazine achieved remarkable alleviation of myocardial tissue damage, with CK, CK-MB, and AST levels reduced to control-comparable levels at both 5 mg/kg and 10 mg/kg doses. This pronounced reduction in myocardial injury markers, coupled with the restoration of myocardial contractility to near-normal levels, suggests that hydralazine prevents cellular injury and subsequently contributes to the recovery of myocardial contractility.

Hematoxylin and eosin (H&E) staining revealed that hydralazine treatment markedly ameliorated the LPS-induced structural disarray, myocardial edema, hemorrhage and most notably, the extensive inflammatory cell infiltration (Figure 4A), suggesting that hydralazine effectively disrupts the inflammatory cell recruitment in myocardial tissue. Moreover, transmission electron microscopy (TEM) observation offered critical insights at the subcellular level, demonstrating that hydralazine significantly attenuated LPS-induced ultrastructural damage, including myofibrillar disarray, mitochondrial swelling, mitochondrial cristae disruption and mitochondrial fragmentation (Figure 4B). The damage to mitochondria may produce excessive reactive oxygen species and cause oxidative injury in myocardial cells [58,59]. Collectively, these histological and ultrastructural observations provide evidence that hydralazine preserves myocardial architecture and cellular organelle function, which underpins the functional recovery observed in echocardiography.

Hydralazine showed a similar myocardial protective effect in the rat myocardial ischemia/reperfusion injury model [21,60]. Notably, previous research has established that inhibition of SSAO by hydralazine, semicarbazide or LJP 1207 can alleviate myocardial ischemia/reperfusion injury in rats [21]. Consequently, these converging lines of evidence from different injury models suggest that hydralazine may confer myocardial protection through a shared mechanism involving SSAO inhibition.

SSAO is critically involved in inflammatory cell recruitment through its dual functions as both an adhesion molecule and an enzyme [61]. The enzymatic activity of SSAO facilitates leukocyte trafficking by promoting their rolling, adhesion, and transendothelial migration, which are the processes essential for inflammatory cell infiltration across endothelium into tissues [62]. While previous studies have reported that LPS can increase SSAO activity systemically [63], herein, we report for the first time that SSAO activity is significantly elevated within the myocardial tissue of septic mice. Hydralazine administration inhibited LPS-induced elevated SSAO activity, concurrently improving cardiac function and survival rates in septic mice, strongly suggesting that SSAO may be involved in the pathophysiological mechanism of SIMD.

Systemically, our results also showed a profound upregulation of the chemokines in septic mice, including CCL1, CCL2, CCL3, etc. Treatment with hydralazine significantly attenuated the upregulation of these chemokines. The reduction in these chemotactic signals provides a mechanistic explanation for the decreased inflammatory cells infiltration observed in myocardial tissue via H&E staining. Chemokines function as central coordinators of leukocyte trafficking, guiding their adhesion to the endothelium and subsequent transmigration into tissues. The observed downregulation of chemokines by hydralazine, coupled with its direct inhibition of SSAO activity, likely disrupts this coordinated recruitment cascade, thus alleviating the inflammatory response in sepsis and promoting immune homeostasis.

Although inflammatory process is considered to play an important role in the pathogenesis of sepsis, the inflammatory response may also be triggered and exacerbated by oxidative stress [64,65]. Elevated lipid peroxidation and decreased antioxidant levels have been observed in patients with sepsis, and similar findings have been consistently replicated in numerous animal models [66,67]. It has been confirmed in both human and animal studies that oxidative stress has a strong association with sepsis and is related to the severity of the disease and mortality [66]. Our results also showed that plasma GSH-PX, T-AOC and SOD activities were significantly decreased in LPS-induced septic mice, and hydralazine intervention significantly improved this situation. GSH-PX and SOD are the key antioxidant enzymes, with GSH-PX catalyzing the conversion of hydrogen peroxide (H_2_O_2_) into water and SOD converting superoxide anion (O_2_^−^) to O_2_ or to the less reactive H_2_O_2_ [65]. T-AOC reflects the overall antioxidant capacity, including both enzymatic and non-enzymatic molecules (such as GSH, metals, vitamins A, C and E) [68]. Therefore, our results indicate that hydralazine significantly enhances the antioxidant defense mechanisms in septic mice, which is crucial because oxidative stress plays a major role in organ dysfunction in septic mice and severity in septic patients as well [65,66]. The reduction in MDA levels and increase in GSH levels in both plasma and myocardial tissue of hydralazine-treated mice further indicated a substantial decrease in oxidative damage. This is in line with our histological observation of myocardial tissue that demonstrated structural destruction of myocardial cells, mitochondrial swelling, and disruption of mitochondrial cristae in septic mice. Administration of hydralazine reversed the effect of LPS on the levels of MDA and GSH, both in plasma and tissues, which may result from the enhancement of antioxidative capacity.

Finally, it is important to acknowledge the limitations of this study. The primary limitation is the use of the LPS-induced endotoxemia model, which represents a highly simplified, acute, and sterile inflammatory response primarily mimicking Gram-negative bacterial infections [69]. Although this model is valuable for its reproducibility and for dissecting initial innate immune and cardiac responses, it does not fully recapitulate the complexity of polymicrobial or clinical sepsis, which often involves a live microbial burden and a more complex interplay between innate and adaptive immunity [70]. A second important consideration for clinical translation is the timing of therapeutic intervention. While our 30 min post-LPS intervention successfully targets the initial hyperinflammatory phase and provides proof-of-concept for the drug’s efficacy, diagnosis and treatment in clinical practice may inevitably occur later, after the first cytokine wave has subsided. Therefore, the efficacy of hydralazine requires further investigation in models with delayed therapeutic intervention. Furthermore, the present study demonstrates a critical alteration in SSAO enzymatic activity without determining its upstream cause (e.g., altered protein expression or post-translational modulation). Beyond SSAO inhibition, the pronounced efficacy of hydralazine suggests that other mechanisms, such as the regulation of cardiomyocyte death pathways (e.g., pyroptosis/ferroptosis) or mitochondrial quality control, may contribute to its protection. Elucidating these mechanisms in future studies will be crucial to fully solidifying the therapeutic repurposing potential of hydralazine for SIMD.

Therefore, while our data provide robust proof-of-concept that SSAO inhibition is a novel and potent strategy against LPS-induced cardiac injury, the therapeutic efficacy of hydralazine must be validated in more clinically relevant models, such as polymicrobial sepsis (e.g., cecal ligation and puncture), and with clinically congruent treatment timelines, before any firm conclusions about its clinical utility can be drawn. Nonetheless, the core pathological mechanisms identified in our study (excessive oxidative stress and inflammatory infiltration) are broadly implicated in septic myocardial injury across various etiologies [71,72]. Thus, therapeutic strategies like hydralazine that target these fundamental processes remain promising for clinical translation, even as their precise efficacy profile requires further elucidation in more complex settings.

## 5. Conclusions

**Figure 9:**
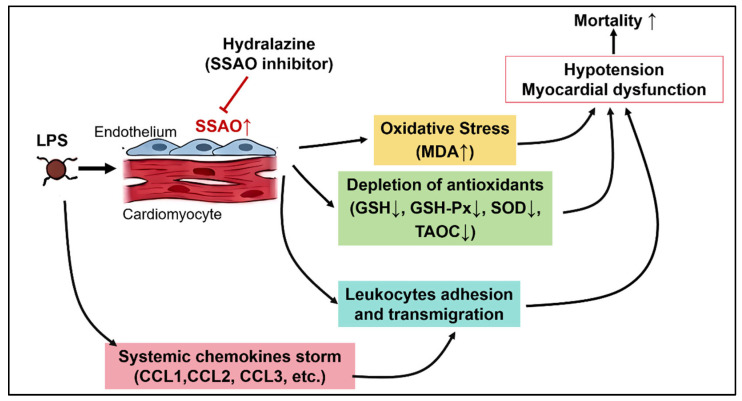
The schematic illustrates the key pathophysiological processes in SIMD and the proposed mechanism of hydralazine. LPS challenge triggers a systemic inflammatory response. This study identifies a novel key event within the heart: the upregulation of SSAO activity. The elevated SSAO activity drives two major pathological pathways: (1) Oxidative Stress: SSAO contributes to oxidative stress by generating reactive oxygen species, leading to lipid peroxidation (increased MDA), depletion of antioxidants (reduced GSH, SOD, GSH-Px), and subsequent mitochondrial damage and cardiomyocyte injury. (2) Inflammation: SSAO/VAP-1 facilitates leukocyte adhesion and transmigration. Concurrently, a systemic chemokine storm promotes inflammatory cell infiltration into the myocardial tissue. These pathological processes collectively lead to myocardial dysfunction, hypotension, and increased mortality. Hydralazine, administered after sepsis onset, acts as a potent inhibitor of SSAO activity. By blocking SSAO, hydralazine attenuates both oxidative stress and inflammatory infiltration, which ultimately preserves cardiac function and improves survival. The “T-bars” indicate the inhibitory action of hydralazine. Arrow directions indicate changes in level: up (↑) for increase and down (↓) for decrease.

In conclusion, our study demonstrates that hydralazine confers significant protective effect against LPS-induced myocardial dysfunction and improves survival rate through a multifaceted mechanism. Hydralazine ameliorates myocardial injury and cardiac contractile dysfunction, attenuates oxidative stress by enhancing antioxidant capacity and reducing lipid peroxidation. Furthermore, hydralazine mitigates the inflammatory response by suppressing chemokine upregulation and subsequent leukocyte infiltration into myocardial tissue. Mechanistically, these protective effects of hydralazine appear to be mediated, at least in part, through the inhibition of SSAO activity, which we identified as being significantly elevated in the septic heart (Figure 9). These findings propose that SSAO inhibition could serve as a novel therapeutic strategy for addressing sepsis-induced myocardial dysfunction and underscore the repurposing potential of hydralazine.

## Figures and Tables

**Table 1:** Severity scoring of myocardial and mitochondrial injury.

Scores	Features
Myocardial injury severity scoring for H&E	
0	Normal myocardium
1	Mild interstitial edema with focal necrosis
2	Moderate myocardial cell swelling and diffuse necrosis
3	Severe ischemia with prominent neutrophil infiltration
4	Extensive damage, characterized by contraction band necrosis, leukocyte infiltration, ischemia, and hemorrhage
Mitochondria injury severity for TEM ^1^	
0	Intact double membrane, compact orderly cristae, and a homogeneous dense matrix
1	Mitochondrial swelling
2	Lysis and breakage of mitochondrial cristae (cristolysis)
3	Mitochondrial matrix proteins disintegrate
4	Large dense granules in the mitochondrial matrix
5	Ruptured and fragmented mitochondria

^1^ Scoring is hierarchical; each grade includes all features of the less severe grades.

## Data Availability

The original contributions presented in this study are included in the article/Supplementary Material. Further inquiries can be directed to the corresponding authors.

## Supplementary Materials

The following supporting information can be downloaded at: https://www.mdpi.com/article/10.3390/antiox14121502/s1, Figure S1: Hydralazine ameliorates physiological deterioration in LPS-induced septic mice; Figure S2: Effect of hydralazine on cardiac function in mice with SIMD during the acute septic shock phase.

## Abbreviations

The following abbreviations are used in this manuscript:

SIMD	Sepsis-induced myocardial dysfunction
SSAO	Semicarbazide-sensitive amine oxidase
LPS	lipopolysaccharide
HYD	Hydralazine
VAP-1	vascular adhesion protein-1
T-AOC	total antioxidant capacity
SOD	Superoxide Dismutase
i.p.	intraperitoneal
LV	left ventricular
LVIDd	The left ventricular internal diameters at end-diastole
LVIDs	The left ventricular internal diameters at end-systole
LVEDV	left ventricular end-diastolic volume
LVESV	left ventricular end-systolic volume
LVSV	left ventricular stroke volume
EF	ejection fraction
FS	fractional shortening
CK	creatine kinase
CK-MB	creatine kinase-MB
LDH	lactate dehydrogenase
AST	aspartate aminotransferase
H&E	Hematoxylin and Eosin
TEM	Transmission electron microscopy
GSH-PX	glutathione peroxidase
MDA	Malondialdehyde
GSH	Glutathione
TBA	2-thiobarbituric acid
CCL	C-C motif chemokine ligand
CXCL	C-X-C motif chemokine ligand
