# Supplementary material for: Hydralazine Attenuates Lipopolysaccharide-Induced Murine Myocardial Dysfunction by Inhibition of Semicarbazide-Sensitive Amine Oxidase
Source: Antioxidants (Basel). 2025 Dec 14;14(12):1502. doi: 10.3390/antiox14121502 (PMC12729300; doi:10.3390/antiox14121502)
Supplement: Supplementary file 1 [file antioxidants-14-01502-s001.zip › antioxidants-3962959-supplementary.pdf]

1. Mice in the control group maintained stable body weight and temperature throughout the observation period. In contrast, Lipopolysaccharide (LPS) administration induced a significant reduction in both body weight and core body temperature (Supplementary Figure S1). Body weight loss reached its nadir on day 4 post-LPS and remained at this low level, while the lowest temperature was observed during the first two days, followed by a slight recovery. Treatment with hydralazine (HYD, 1-20 mg/kg) significantly attenuated the LPS-induced hypothermia within 2 days and ameliorated the body weight loss by day 4, in a dose-dependent manner.

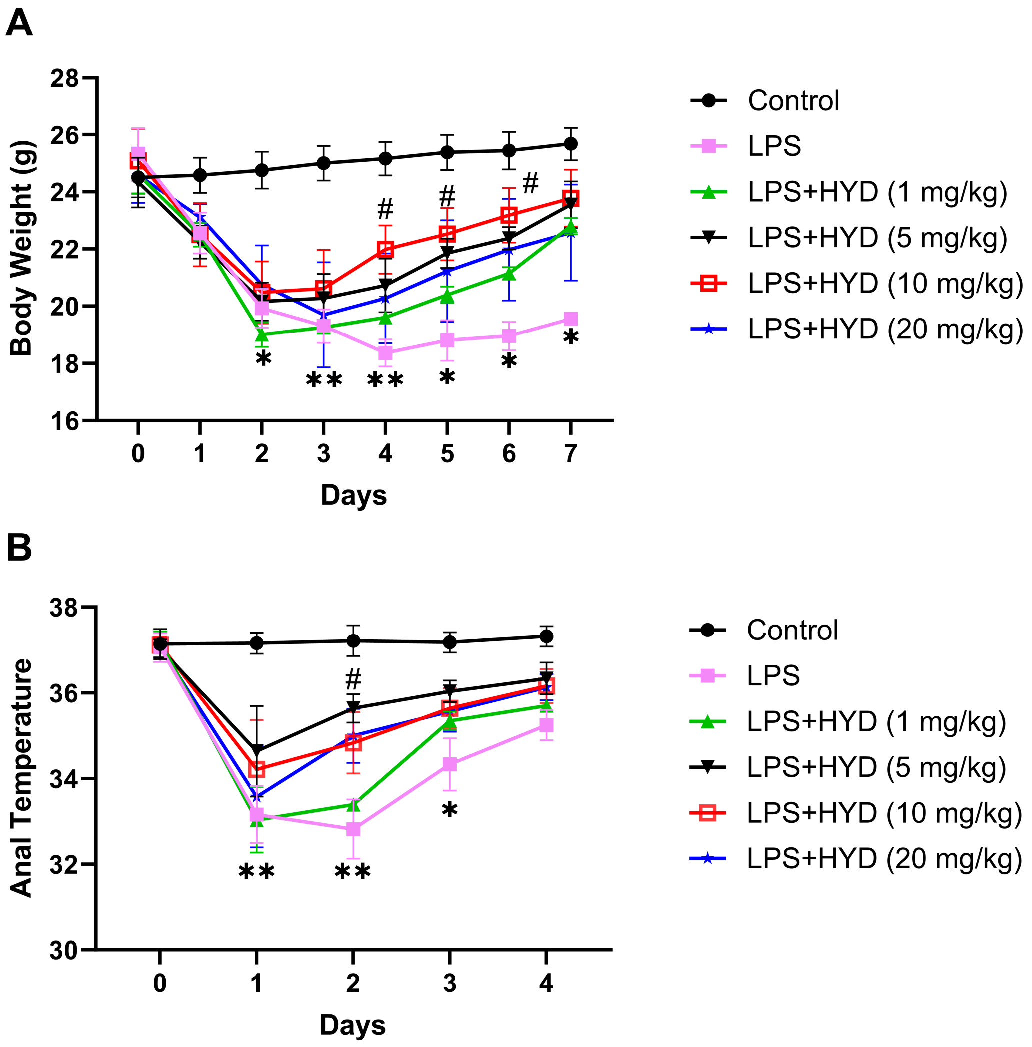

Figure S1. Hydralazine ameliorates physiological deterioration in LPS-induced septic mice.

(A) Changes in body weight and (B) Anal temperature were monitored in mice from the survival study groups (n = 10) following LPS or saline injection at Day 0. Data are presented as mean ± SD. *p < 0.05, **p < 0.01, vs. Control group; # p < 0.05 vs. LPS group (by two-way ANOVA with Tukey's post-

hoc test).

2. Representative M-mode echocardiograms for all experimental groups across key time points (baseline, and 2, 6, 12, 24 hours post-LPS) are provided in Supplementary Figure S2.

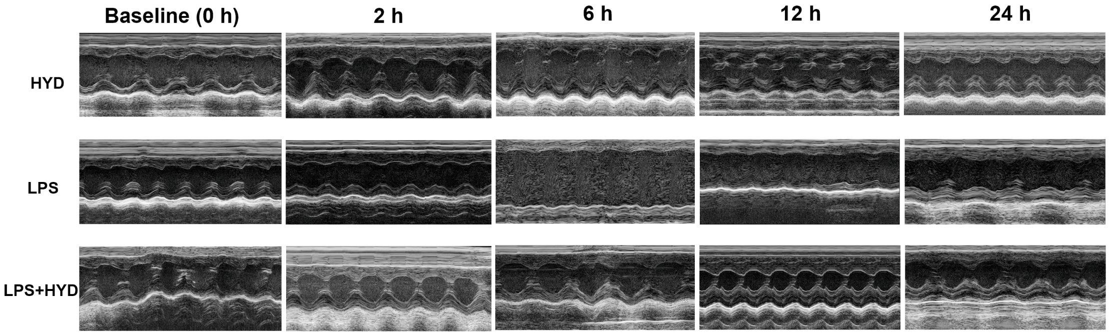

Figure S2. Effect of hydralazine on cardiac function in mice with SIMD during the acute septic shock phase. Representative M-mode echocardiograms from HYD-only (10 mg/kg), LPS, and LPS+HYD (10 mg/kg) groups at baseline (0 h) and at 2, 6, 12, and 24 hours after LPS injection. The images visually demonstrate the stable cardiac function in HYD groups over time. In contrast, the LPS group shows a progressive deterioration in contractility (evident as reduced wall motion and increased left ventricular chamber size during systole), which is most severe at the 12-hour time point. Treatment with hydralazine (LPS+HYD group) markedly preserved systolic function throughout the observation period, as seen by the maintained wall thickening and reduced left ventricular dimensions during systole, comparable to the baseline.
